# Enhancing Mid-Term Strength and Microstructure of Fly Ash–Cement Paste Backfill with Silica Fume for Continuous Mining and Backfilling Operations

**DOI:** 10.3390/ma17246037

**Published:** 2024-12-10

**Authors:** Xiaoping Shao, Zhengchun Wang, Renlong Tang, Bingchao Zhao, Jianbo Ning, Chuang Tian, Wei Wang, Yibo Zhang, Xing Du

**Affiliations:** 1 Energy School, Xi’an University of Science and Technology, Xi’an 710054, China; shaoxp@xust.edu.cn (X.S.); 22203077033@stu.xust.edu.cn (Z.W.); zhaobc913@163.com (B.Z.); 21203077026@stu.xust.edu.cn (J.N.); 20203077027@stu.xust.edu.cn (C.T.); 17203078034@stu.xust.edu.cn (W.W.); 23203226112@stu.xust.edu.cn (Y.Z.); 23203226054@stu.xust.edu.cn (X.D.); 2 Key Laboratory of Western Mines and Hazards Prevention, Ministry of Education of China, Xi’an 710054, China

**Keywords:** continuous mining and continuous backfilling, silica fume, fly ash–cement paste filling material, mechanical properties, microstructure

## Abstract

Fly ash–cement composite backfill slurry, prepared by partially replacing cement with fly ash, has been demonstrated to effectively reduce the mine backfill costs and carbon emissions associated with cement production. However, the use of fly ash often results in insufficient early and medium-term strength of the backfill material. To address the demand for high medium-term strength in backfill materials under continuous mining and backfilling conditions, this study developed a silica fume–fly ash–cement composite backfill slurry. The effects of varying silica fume contents on the slurry’s flowability, uniaxial compressive strength, microstructure, and pore characteristics were systematically investigated. The results showed that increasing the silica fume content significantly reduced the slurry’s flowability. However, at a silica fume content of 5%, the slurry achieved optimal medium-term strength, with a 14-day uniaxial compressive strength of 3.98 MPa, representing a 25% improvement compared to the control group. A microstructural analysis revealed that a moderate silica fume content promoted the formation of calcium silicate hydrate gel, filled micropores, and optimized the pore structure, thereby enhancing the overall strength and durability of the material. Conversely, an excessive silica fume content above 5% led to a marked decrease in both flowability and strength. Based on a comprehensive evaluation of silica fume’s effects on the flowability, strength, and microstructure, the optimal silica fume content was determined to be 5%. This study provides a theoretical basis and practical guidance for improving the efficiency of continuous mining and backfilling operations, and for designing high-performance backfill materials suitable for continuous mining and filling conditions.

## 1. Introduction

China possesses abundant coal resources. According to a forecast, in 2050, the percentage of coal in China’s energy composition will persist at approximately 50% [1]. China’s “three under” coal reserves consist of more than 15 billion tons, resulting in a large number of coal resources that have not been fully exploited and face the problem of waste [2]. The proposed filling mining technology effectively solves the problems of surface subsidence, water damage, and solid waste treatment caused by ‘three under’ coal mining [3,4].

The continuous mining and continuous backfilling (CMCB) mining method greatly improves mining efficiency by realizing the non-interference and continuous parallel operation of filling and mining [5]. Continuous mining and continuous backfilling adopts the process flow of block mining and filling cycle operations. To ensure the filling effect, the width of each section is maintained at about 150 m. In the process of the mining and filling cycle, the medium-term strength of the filling body has an important influence on the continuation rate of the working face, while the later strength plays a key role in the control of surface subsidence after full mining and full filling in the later stage of mining [6].

However, especially in the use of continuous mining and continuous filling to recover irregular coal bodies such as triangular coal and roadway coal pillars [7], the width of the coal body may be far less than 150 m, and the problem of continuous mining and continuous backfilling is extremely prominent. To improve the continuous efficiency of continuous mining and continuous filling of the working face, prevent a disconnection between mining and filling, and achieve the purpose of improving the production efficiency, safety, and environmental protection effect of the continuous mining and continuous filling process [8], the key to solving this bottleneck problem is to develop a filling body that effectively improves the medium-term strength of the filling body and does not reduce the later strength [9].

Cement is one of the most commonly used materials for preparing filling slurry in construction and mining applications [10,11]. However, the production of cement clinker is associated with significant carbon dioxide emissions and high costs [12,13]. To address these challenges, fly ash (FA), a byproduct of coal combustion, has emerged as a sustainable and cost-effective alternative. By the end of 2024, China’s fly ash emissions are expected to reach 930 million tons, providing an abundant resource to replace cement, particularly in the preparation of fly ash–cement paste (FCP) for backfill mining operations [14]. Despite its widespread industrial application, fly ash has raised concerns about its potential radioactivity due to the presence of naturally occurring radionuclides such as radium-226 (^226^Ra), thorium-232 (^232^Th), and potassium-40 (^40^K). The activity concentrations of these radionuclides vary depending on the coal source and combustion conditions. In this study, the analyzed fly ash samples exhibited activity concentrations of ^226^Ra 66.8 Bq/kg, ^232^Th 149 Bq/kg, and ^40^K 790 Bq/kg [15]. According to UNSCEAR recommendations [16], the activity limits for these radionuclides are as follows: ^226^Ra: <200 Bq/kg, ^232^Th: <250 Bq/kg, ^40^K: <1000 Bq/kg. The fly ash samples in this study fell well within these acceptable limits. However, compared to the average activity concentrations in Chinese soil (^226^Ra: 36.5 Bq/kg, ^232^Th: 49.1 Bq/kg, ^40^K: 580 Bq/kg) [15], the radioactivity of fly ash is higher. Nonetheless, the radium equivalent activity (Raeq) of the fly ash samples was calculated to be 335 Bq/kg, which is below the international safety threshold of 370 Bq/kg. This indicates that fly ash is generally safe for industrial applications, such as in construction materials and backfill [17]. However, in applications with stricter environmental requirements, such as residential or agricultural areas, variations in radioactivity levels may limit the broader use of fly ash. Studies show that the radioactivity levels of fly ash samples in China are typically within acceptable ranges for industrial applications, particularly in construction and mining backfill operations, where radiological risks are low and manageable. The fly ash samples used in this study were sourced from the filtration system of the Boluo coal-fired power plant in Hengshan County, Shaanxi Province, China. Their radiological data indicate that, under strict management, fly ash is a viable alternative to cement. Overall, fly ash serves as a sustainable substitute that not only reduces carbon dioxide emissions but also significantly lowers backfill costs while meeting safety requirements for most industrial applications.

FA has many advantages as a cementitious material itself: the proportion of active SiO_2_ and Al_2_O_3_ in fly ash is large, and the pozzolanic effect improves the strength of the filling body [18]; FA particles are usually spherical in shape, which helps to provide a ‘ball’ function and enhance the viscosity of the slurry [19]. Liu [20] found that FA molecules remain inactive during the initial hydration process, resulting in a lower degree of early hydration reaction and affecting the early strength of the filling body. Based on the above research, we can see that although FA has been extensively utilized in the domain of filling mining, the inadequacy of its early hydration reaction leads to the insufficient strength of FCP in the early and middle stages [21,22], making it difficult to meet the requirements of CMCB for the medium-term strength of the filling body. Consequently, it is essential to identify a substance that can be used in combination with FCP to improve the medium-term strength of the filling body and improve the production efficiency of CMCB.

Silica fume (SF) is obtained by collecting and purifying tail gas in the process of smelting silicon-containing products [23]. In 2023, China’s industrial silicon production was estimated to reach 3.25 million tons [24,25,26], but emissions are far greater than the amount that is utilized [27,28], taking up a lot of land resources. The use of SF in backfill operations not only reduces cement consumption, significantly lowering carbon emissions, but also promotes resource recycling. These contributions position silica fume as a key component in advancing the long-term sustainability of continuous mining operations. Mojtaba Nili [29] used concrete containing SF to perform best in the salt scale test. The incorporation of SF decreased the overall pore volume and optimized the pore distribution. Ni Chenxin [30] studied the effect of SF fineness on the hydration process of cement paste. An increase in the SF particle size extends the acceleration period of cement hydration and reduces the heat release rate. Hengrui Liu [31] found that the particles of SF are very fine, which increases the proportion of fine particles in the material, enhances water absorption, and diminishes the fluidity of the slurry. Jeong [32] found that SF particles have a high designated surface area, which can offer nucleation sites for the hydration process of cement, and SF has a high content of SiO_2_ active components, which can interact with Ca(OH)_2_ generated by cement hydration.

Based on the above research, we can see that SF in cement-based materials improves the porosity, hydration rate, etc. [33]. The high volcanic ash content of SF makes up for the problem of insufficient early activity of FCP, while the larger particle size of FA can solve the problem of the slurry fluidity reduction caused by the extremely fine SF particles. Therefore, this study mainly takes the medium-term strength of SFCP as the research object, replacing cement with different amounts of SF to join FCP and prepare silica fume–fly ash–cement composite filling paste (SFCP) to solve the problem of the low medium-term strength of FCP. The existing research on backfill materials has primarily focused on optimizing flowability and strength under conventional conditions. In contrast, studies related to continuous mining and backfilling have mainly concentrated on overburden movement and mechanical transfer mechanisms. Research specifically addressing the performance of backfill materials under the unique conditions of continuous mining and backfilling remains relatively scarce [34].

In this study, the application effect of five kinds of cement with equal mass replacements of SF in improving the medium-term strength of the filling body is examined. The effects of different SF contents on the flow characteristics, mechanical properties, hydration products, porosity, and microstructure evolution of FCP are analyzed by means of fluidity, uniaxial compressive strength, and microstructure tests. This provides a theoretical foundation and practical guidance for the optimization design of backfill materials under continuous mining and backfilling conditions.

## 2. Materials and Methods

### 2.1. Experimental Materials

All materials were subjected to a standard drying treatment prior to analysis. Specifically, the samples were dried in an oven at 105 ± 5 °C for 12 h until a constant weight was achieved. The sampling process followed the stratified sampling method, with multiple samples collected, mixed, and subsequently divided to obtain a homogeneous sample. After drying, the samples were sealed and stored in a desiccator for subsequent analysis.

#### 2.1.1. Silica Fume

**Figure 1:**
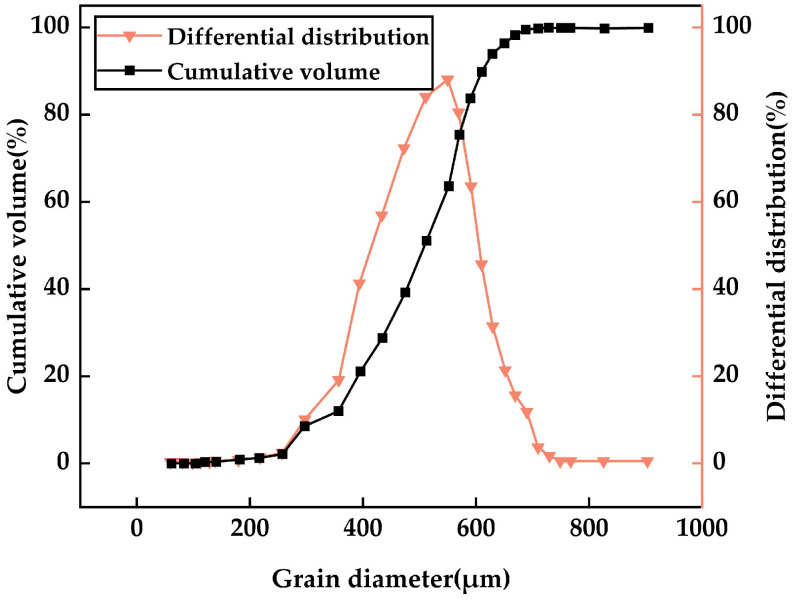
Particle size distribution of SF.

In this study, silica fume (SF) was taken from the fine micro-silicon powder developed by Sanyuan Company in Gansu Province, China. The bulk density was 320 kg/m^3^ and the specific surface area was 18,500 m^2^/kg. Figure 1 shows the particle size distribution of the SF. The particle size distribution of silica fume samples was determined using a Malvern Mastersizer 3000 laser particle size analyzer (Malvern Panalytical, Malvern, Worcestershire, UK), which has a measurement range of 0.01–3000 µm in wet dispersion mode. The samples were dispersed in deionized water, with 0.2–0.5 wt% sodium hexametaphosphate added as a dispersant. Ultrasonic treatment was applied for 15 min to ensure a uniform particle dispersion. The measurements were repeated three times, and the particle size distribution curve, along with the relevant parameters, is reported. It was found that the maximum particle size of the SF was less than 1 μm, and the average particle size was 0.1 μm. The SF predominantly consisted of silica (90.36 wt%). The primary chemical makeup of the SF is presented in Table 1.

#### 2.1.2. Cement

Ordinary Portland cement (OPC, P.O.42.5R) was used to make SFCP samples. Ordinary Portland cement is one of the most commonly used materials in mine functional filling and is the main component providing compressive strength. The main chemical composition of OPC is shown in Table 1.

#### 2.1.3. Gangue

The gangue came from Shenmu City, Shaanxi Province. The gangue was used as an aggregate, and the main components were SiO_2_, CaO, Al_2_O_3_, K_2_O, and Fe_2_O_3_. Table 1 presents the main chemical composition of the gangue.

#### 2.1.4. Fly Ash

**Figure 2:**
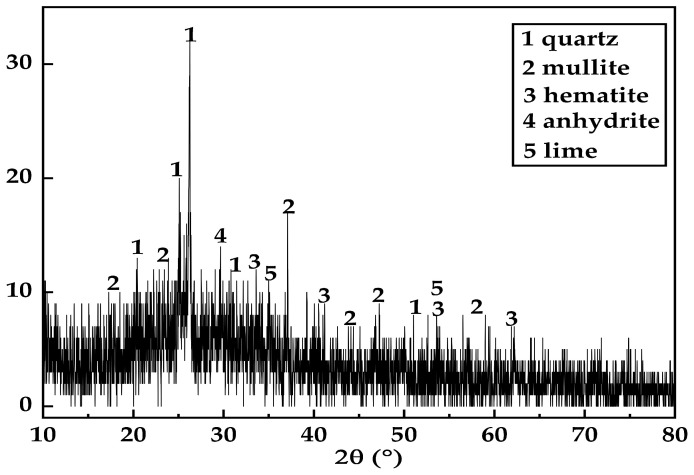
XRD results for fly ash.

The fly ash came from Shenmu City, Shaanxi Province, China. According to the current Chinese standard Technical Specification for the Application of Fly Ash in Cement and Concrete (GB/T 1596-2017) [35], the fly ash recycling methods discussed in this study meet the requirements for relevant chemical and physical properties. Its appearance was that of a gray-black powder. The specific surface area was 11.8 m^2^/kg. The main components were SiO_2_, CaO, Al_2_O_3_, K_2_O, and Fe_2_O_3_, of which CaO accounted for 8.11%. These compounds enhance the durability of the filler material [36]. The XRF test results for the FA are shown in Table 1. Figure 2 presents the XRD pattern of the FA.

### 2.2. Sample Preparation

In this experiment, to study the effect of SF on the UCS of SFCP samples, we used the same quality SF to replace the cement; the replacement ratio was 0.0% (control test group) 2.5%, 5%, 7.5%, or 10%, numbered C-SF0, C-SF2.5, C-SF5, C-SF7.5, or C-SF10, respectively. Using a solid mass concentration of 76%, the materials, such as FA and SF, were fully stirred, and then water was added to fully stir for more than 5 min. At the same time, a bar was inserted into the mixture to eliminate bubbles and fill the voids. Then, the stirred mixture was poured into a plastic mold with a height of 150 mm and a diameter of 50 mm. The mixture was left to solidify, the mold was removed, and the SFCP sample was prepared. The prepared SFCP samples were positioned in a typical curing box for the curing process, and the corresponding curing ages were 3, 7, 14, and 28 days. Table 2 presents the ratios of the test materials.

### 2.3. Experimental Methods

#### 2.3.1. Setting Time Test

The total length of the blade was (165 ± l) mm, the outer diameter of the blade shaft was (20.0 ± 0.5) mm, and the diameter of the positioning hole was about 12 mm. The measurement method was as follows: The cement slurry specimen was prepared under the standard consistency water consumption, and the first measurement was started after curing in the standard curing box for 30 min. The test die was transferred to the fixed center of the Vicat instrument, and the screw was tightened for 1~2 s to make the test rod sink vertically. The distance was recorded after sinking or releasing for 30 s. After the initial setting time test, the specimen was inverted 180° and positioned in a normal curing box for maturation. The specimen was transferred to the fixed center of the Vicat instrument, and the screw was tightened for 1~2 s so that the test rod sank vertically. When the test needle penetrated the test body by less than 0.5 mm, it was in the final solidification state.

#### 2.3.2. Analysis of Liquidity

A support expansion measurement base and a micro-shrinkage cylinder with top and bottom diameters of 50 mm and 100 mm and a height of 150 mm were used for testing. Each micro-slump experimental group was replicated three times to compute the average micro-slump value, thus ensuring the scientific validity of the experimental results.

Rheological experiments were conducted to measure the rheological curves of SFCP samples with different proportions using a Viscotester iQ rotational rheometer (Thermo Fisher Scientific, Waltham, MA, USA). The rheometer had a rotational speed range of 0.01–1500 rad/s and a torque sensitivity of 0.01 nNm. During the 180 s test, the shear rate was increased from 0.1 s^−1^ to 100.1 s^−1^. A coaxial blade rotor was employed to plot and measure the rheological curves. The company’s headquarters is located in Waltham, MA, USA, and the equipment was manufactured in Karlsruhe, Germany. The data were processed using HAAKE RheoWin software, version 3.83.0003.

#### 2.3.3. Uniaxial Compressive Strength Test

According to the ASTM C39 (ASTM C39/C39M-18, 2024) [37] standard, the uniaxial compressive strength test was carried out on SFCP samples with curing ages of 3, 7, 14, and 28 days to detect their compressive strength. Firstly, the sample was installed between the two plates of the DNS-100 electronic universal testing machine to ensure that the two ends of the SFCP sample were smooth, and the compression test was carried out. Each specimen was loaded at a deformation rate of 1 mm/min. The maximum stress value (failure peak) was determined as the UCS value during the compression test, and the average value of the three strengths was used as the final UCS value of the group.

#### 2.3.4. Microstructure Analysis

To analyze the effect of SF on the strength development of SFCP samples from the microstructure, scanning electron microscope (SEM), X-ray diffraction (XRD), and porosity tests were carried out. After the completion of the UCS test, some sample centers were taken for mineral composition tests and micro-morphology analysis. 

Initially, the hydration reaction of the samples was terminated using anhydrous ethanol. The hydrated samples were then dried in a 50 °C oven for 24 h. After drying, the samples were tested using SEM. The morphology and crystal structure of the hydration products in the SFCP samples were observed through SEM analysis. A Quanta 250 FEG scanning electron microscope (FEI Company, Hillsboro, OR, USA) was used, with a wide accelerating voltage range of 200 V to 30 kV and a maximum beam current of 200 nA. The analysis was conducted using xT Microscope Control software, version xTUI 3.0. The instrument scanned the internal texture, dimensions, and shapes of the hydration products at an accelerating voltage of 30 kV and a resolution of 1.0 nm.

Additionally, the types and diffraction intensities of hydration products in the SFCP samples were analyzed using XRD. The experiment utilized an X’Pert PRO MPD diffractometer manufactured by PANalytical in Almelo, The Netherlands. The instrument featured a maximum power of 3 kW, a maximum tube voltage of 60 kV, and a maximum tube current of 55 mA. In this experiment, the scanning speed was set at 10° per minute, covering a 2θ range from 10° to 80°. A data analysis was conducted using X’Pert HighScore Plus software, version 5.2.

In the mercury intrusion porosimetry (MIP) test, a Micromeritics Autopore V9620 was used for analysis. This equipment is manufactured by Micromeritics Instrument Corporation, based in Norcross, GA, USA. The analysis was performed using MicroActive software, version V5.9.

The sample was first weighed to obtain its mass (M1). A suitable dilatometer was selected, into which the sample was poured, sealed, and weighed to determine the combined mass of the sample and dilatometer (M2). The dilatometer was then placed in the low-pressure station to prepare for low-pressure analysis. After the low-pressure analysis was completed, the dilatometer was removed and weighed. Subsequently, the dilatometer was transferred to the high-pressure station to begin high-pressure analysis. The exhaust valve of the high-pressure station was operated as instructed during the process.

**Figure 3:**
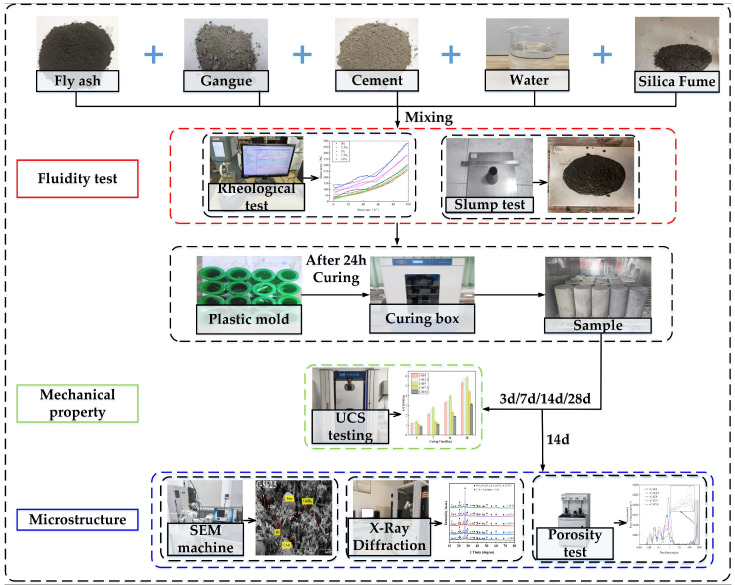
Experimental flow chart.

In this study, a variety of experimental methods were used to explore the performance changes of SFCP under the influence of SF, and the effects of different amounts of SF on the basic properties of the filling materials were analyzed. Figure 3 shows the whole process of the experiment in detail in the form of a flow chart.

## 3. Results and Discussion

### 3.1. Effect of SF on SFCP Setting Time

**Figure 4:**
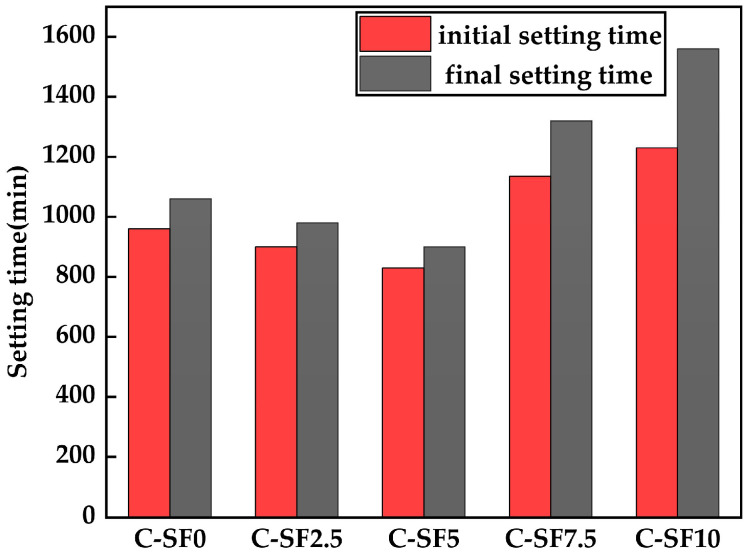
The initial and final condensation times of SFCP.

Figure 4 shows the condensation times (initial condensation times and final condensation times) of the five groups of SFCP. It can be seen from Figure 4 that with the gradual increase in SF incorporation, the initial setting time and final setting time show a trend of being first shortened and then prolonged. It is important to observe that when the SF concentration is regulated at 5% or lower, its influence on the setting time is relatively limited and does not cause significant changes. However, once the content of SF exceeds this range, the setting time of the SFCP is significantly prolonged. The initial setting times of the five groups of SFCP slurry were between 830 min and 1235 min, and the final setting times were between 900 min and 1560 min. The initial coagulation time and final coagulation time of the control group (C-SF0) were 960 min and 1060 min, respectively. The initial setting time and final setting time of C-SF5 slurry were the shortest, at 900 min and 980 min, respectively. The condensation rate increased by 6.25% and 7.5%, respectively, compared with the control group. In the case of only a change in the SF content, the reason for this situation may be that when the addition rate of SF is not more than 5%, it can promote the hydration process of cement, lead to an increase in the internal temperature of hardened paste, reduce its internal humidity, evaporate free water in capillary pores, and cause the dry shrinkage of samples, thus accelerating the setting. The initial setting time and final setting time of the slurry of C-SF10 were the longest, reaching 1235 min and 1360 min, respectively, and the setting speed was reduced by 28.6% and 28.3%, respectively, in comparison to the control group. This is because SF replaced too much cement, resulting in the hydration capacity of the cement itself being affected; additionally, SF adsorbs a large amount of water, seriously hindering the hydration reaction process of cement [38]. Some scholars have also found that in the nano-silica and fly ash cement system, the setting time decreases slightly with the addition of less than 6% SF [39].

### 3.2. Analysis of the Influence of SF on SFCP Liquidity

#### 3.2.1. Analysis of the Effect of SF on the Diffusion and Slump of SFCP

**Figure 5:**
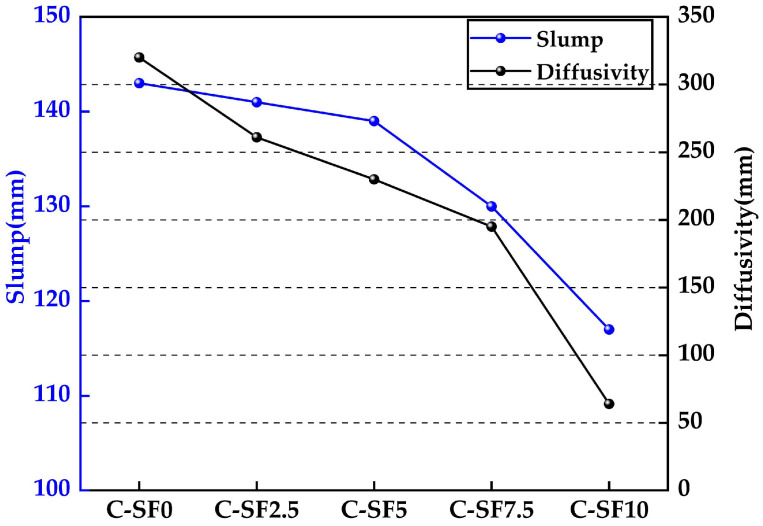
Slump and diffusivity of fresh SFCP slurry.

Figure 5 shows a comparison of the diffusion and slump of the five groups of SFCP. Through the observation of Figure 5, we can see that with the gradual increase in the SF content, the diffusivity and slump show a continuous decreasing trend, indicating that an increase in the SF content significantly reduces the fluidity of SFCP. This phenomenon is particularly evident in C-SF7.5 to C-SF10, indicating that 7.5% may be a critical point; that is, beyond this content, the negative effect of SF on SFCP fluidity is aggravated. The diffusivity of C-SF0 was 320 mm, the diffusivity of C-SF5 was 230 mm, and the diffusivity was reduced by 28%. The slump of C-SF0 was 93 mm, the slump of C-SF5 was 89 mm, and the slump was reduced by 4%. This effect may be attributed to the increased specific surface area of SF, which results in a gradual rise in the amount of water molecules adsorbed on its surface, which strengthens the pozzolanic effect and microcrystalline nucleation of SF. These factors jointly promote the hydration process of SF, strengthen the chemical transition bonds between particles, and produce van der Waals forces. These changes together accelerate the hydration rate of the slurry, improving its consistency, and the slump and diffusivity are thereby reduced to varying degrees [40]. In particular, from C-SF7.5 to C-SF10, the diffusivity decreased from 195 mm to 64.3 mm, a decrease of 67%. The slump decreased from 80 mm to 67 mm, a decrease of 16%. The water absorption effect of SF became more obvious, absorbing a large amount of water, causing the slump and diffusivity of the SFCP to decrease sharply. This phenomenon is consistent with the results of Cairong Luu [41]: when the amount of added SF was greater than 8%, the slump decreased significantly with an increase in SF. However, their study examined different cement contents, aeolian sand contents, and SF contents, indicating that SF has the same effect on slump for different cementitious materials.

The slump test is a critical indicator for evaluating the performance of slurry, as it not only reflects the cohesion and shear resistance of the slurry but also serves as a fundamental parameter for assessing its pumpability. According to relevant studies, the minimum slump value for a slurry is 44.4 mm, while a slump exceeding 70.8 mm is considered indicative of good flowability [42]. Based on this standard, the slump values of the five tested samples in this study all meet the requirements for pipeline transportation, demonstrating excellent pumpability.

#### 3.2.2. Effect of SF on Rheological Properties of SFCP

**Figure 6:**
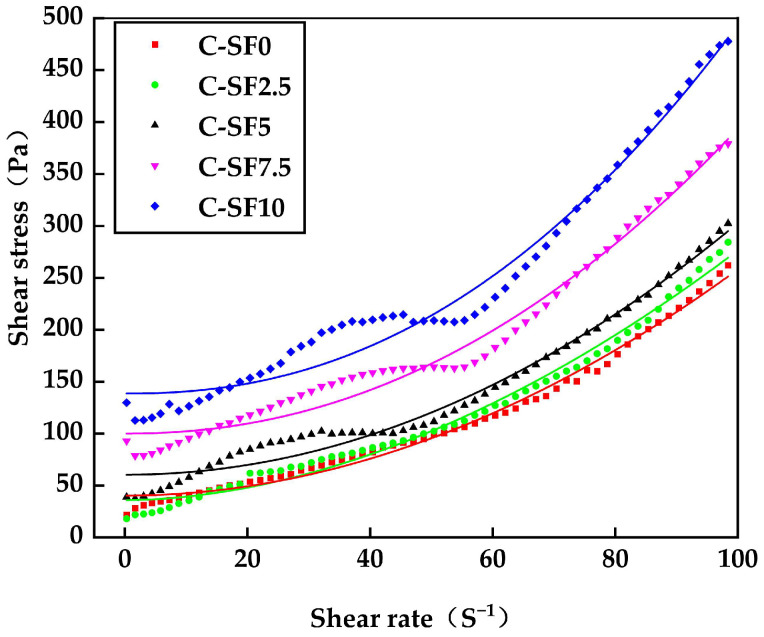
Shear stress of SFCP slurry.

**Figure 7:**
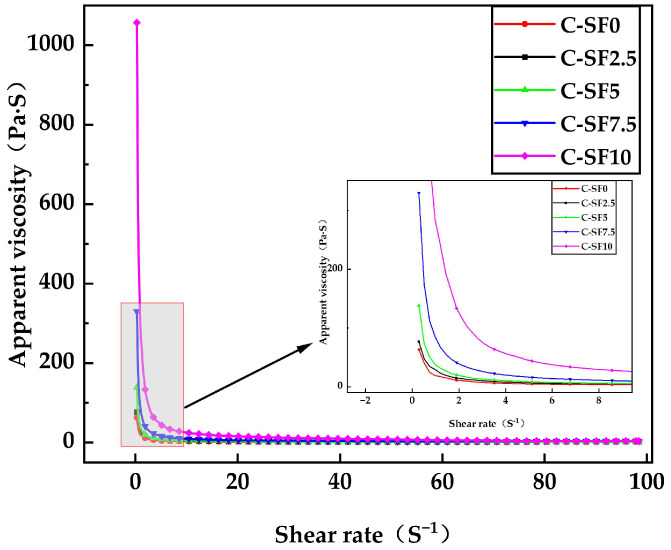
Apparent viscosity of SFCP slurry.

Figure 6 and Figure 7 show the rheological properties of SFCP slurry with different SF contents. The fitting results and rheological parameters are presented in Table 3. It can be seen from Figure 6 that the SFCP slurry is a non-Newtonian fluid, and the shear stress increases with an increase in the shear rate, the different colored lines represent fitting curves derived using Herschel-Bulkley Model to analyze the shear stress and shear rate relationship of SFCP slurry samples. With the increase in the SF content, the shear stress and yield stress increase significantly. At an equivalent shear rate, an increased SF content is associated with greater shear stress of the sample, signifying that the use of SF changes the volume and distribution of solid particles, making the whole system more compact and significantly improving the viscosity of the material. From C-SF0 to C-SF5, the yield stress increased from 35.97 Pa to 60.45 Pa, with a growth rate of 68%. This condition arises because SF, characterized by a large specific surface area, is substituted for cement, which has a smaller specific surface area. Consequently, the overall specific surface area of the particles in the slurry increases, leading to a heightened water demand and an escalation in yield stress [43]. In addition, as the SF content increases, the content of fine particles in the slurry increases significantly, and the water absorption also increases significantly. The free water that plays a lubricating role is adsorbed by SF, which reduces the thickness of the lubricating film between the particles [44]. Therefore, in the process of gradually increasing the content of SF, the yield stress of the slurry is significantly increased when it is deformed by shear force.

Figure 7 illustrates that the plastic viscosity of SFCP slurry diminishes as the shear rate increases across various SF concentration values, showing the shear thinning phenomenon. With an increase in the shear rate, the shear force is sufficient to overcome the interaction between the particles, the particles begin to be rearranged or dispersed, the internal friction of the fluid decreases, and the viscosity decreases. Especially for C-SF10, this agglomeration effect is more obvious; it exhibits a high initial viscosity, but once the structure is destroyed, the viscosity decreases rapidly. From C-SF0 to C-SF5, the variation in SF is 5%, and the maximum apparent viscosity increases from 63.39 Pa to 138.4 Pa, with a growth rate of 118.3%. This is because the network structure produced by cement hydration is destroyed due to the shear force on the SFCP slurry, and the particles are rearranged under the action of this shear force. On the one hand, the shear stress destroys the initial network structure of the filling slurry, resulting in a decrease in the plastic viscosity of the filling slurry. On the other hand, the hydration products produced by cement hydration in the filling slurry gradually form a network structure, leading the formed network structure and shear stress to reach a certain balance so that the plastic viscosity of the slurry first decreases and then remains stable. In addition, SF can promote the hydration process of cement, generate gel products, and enhance the cohesion between particles. The friction among particles, the stiff interconnection of solid particles, and colloidal interactions are the determinants of yield stress [45]. From C-SF7.5 to C-SF10, the change in SF is only 2.5%, and the maximum apparent viscosity reaches 1057 Pa from 329.9 Pa, with a growth rate of 220.4%. This may be because SF reaches its critical concentration, the van der Waals force and electrostatic interaction force between particles are enhanced, and the stronger agglomeration effect leads to a significant increase in the interaction and cohesion between particles. The resistance inside the SFCP slurry increases sharply, thus greatly increasing the apparent viscosity.

To address the observed issues of reduced flowability and increased viscosity at higher SF contents, potential optimization strategies were investigated. The study revealed that maintaining the SF content at 5% can maximize the medium-term strength while maintaining reasonable flowability. Conversely, when the SF content exceeds 7.5%, the flowability and slump values decrease significantly. Therefore, it is recommended to limit the SF content to 5% in practical engineering applications to achieve a balance between viscosity and flowability. Additionally, increasing the water-to-binder ratio and introducing high-performance superplasticizers were found to significantly improve the slump flow and reduce viscosity, further enhancing the flowability characteristics.

### 3.3. Effect of SF on Uniaxial Compressive Strength of SFCP

**Figure 8:**
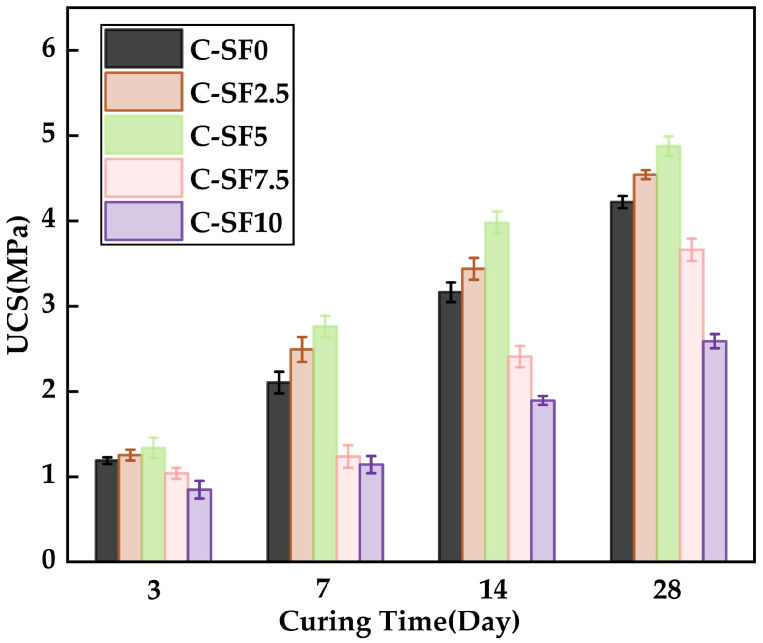
UCS of SFCP at four ages.

The variation in the UCS of SFCP samples with time under different SF additions is shown in Figure 8. From Figure 8, it can be seen that the addition of 2.5% to 5% SF in the SFCP samples significantly improved their UCS, which fully proves the positive role of SF in enhancing the UCS of FCP materials. However, when the content of SF exceeded 5%, it was observed that the UCS value was inferior to that of the control group during the whole period, which indicates that excessive SF has a significant negative effect on the UCS of SFCP. C-SF5 showed the best UCS during the whole period. At 14 days, the UCS of C-SF0 was 3.16 MPa, while the UCS values of C-SF2.5 and C-SF5 were 3.43 MPa and 3.98 MPa, respectively, with growth rates of 8% and 25%, respectively. The UCS values of C-SF7.5 and C-SF10 were 2.41 MPa and 1.896 MPa, respectively, reduced by 23% and 41%, respectively. The reason for the increase in the UCS in the SF2.5 and C-SF5 groups is that, on the one hand, the SiO_2_ in SF reacts with OH- to break the Si-O bond. Because SF has high activity and is rich in SiO_2_, large numbers of broken Si-O bonds are more likely to react with C-H gel to form C-S-H gel. On the other hand, the extremely small SF particles can effectively fill the small cracks in the SFCP samples [46]; this makes the internal structure of the sample more dense, so the compressive strength is improved accordingly [47]. Therefore, C-SF5 has higher UCS.

Similarly, at 14 days, when the SF content was gradually increased to 7.5% and 10%, the UCS values of C-SF7.5 and C-SF10 were 2.34 MPa and 1.89 MPa, respectively, and were 28% and 42% lower than that of C-SF0, respectively. The reason for this situation is that with the increase in SF instead of cement, the production of Ca(OH)_2_ in the process of cement hydration decreases. The decrease in Ca(OH)_2_ makes the amount of OH- ions required for the pozzolanic reaction of SF insufficient. Excessive SF molecules are not easy to diffuse uniformly, and the agglomeration of SF increases the adsorption of water and directly hinders the hydration process of FA and cement. In the cement system, improvement of the UCS depends on the formation of C-S-H, rather than an excessive dependence on the amount of particle filling. Several scholars have employed the amalgamation of alkali-activated slag and SF in their studies and also observed that UCS exhibits an initial increase followed by a subsequent decrease when the SF content is increased [48].

To investigate the effect of different SF contents (0%, 2.5%, 5%, 7.5%, 10%) on the compressive strength, a one-way analysis of variance (ANOVA) was conducted for significance testing. The relevant calculation steps are detailed in Equations (1)–(8). To ensure clarity and conciseness, the detailed calculation process is not elaborated in the main text. The final test results are presented in Table 4, while the corresponding symbols are summarized in the Symbol Table.

(1) $$\bar{X}=\frac{\sum_{i=1}^{15}X_{i}}{15}$$

(2) $$SSB=\sum_{i=1}^{k}n_{i}{\left({\bar{X}}_{i}-\bar{X}\right)}^{2}$$

(3) $$SSW=\sum_{i=1}^{k}\sum_{j=1}^{n_{i}}{\left({\bar{X}}_{ij}-{\bar{X}}_{i}\right)}^{2}$$

(4) $$df_{between}=k-1$$

(5) $$df_{within}=\sum_{i=1}^{k}\left(n_{i}-1\right)$$

(6) $$MSB=\frac{SSB}{df_{between}}$$

(7) $$MSW=\frac{SSW}{df_{within}}$$

(8) $$F=\frac{MSB}{MSW}$$

### 3.4. Microstructure Analysis of SFCP

#### 3.4.1. X-Ray Powder Diffractometer Image Analysis of SFCP

**Figure 9:**
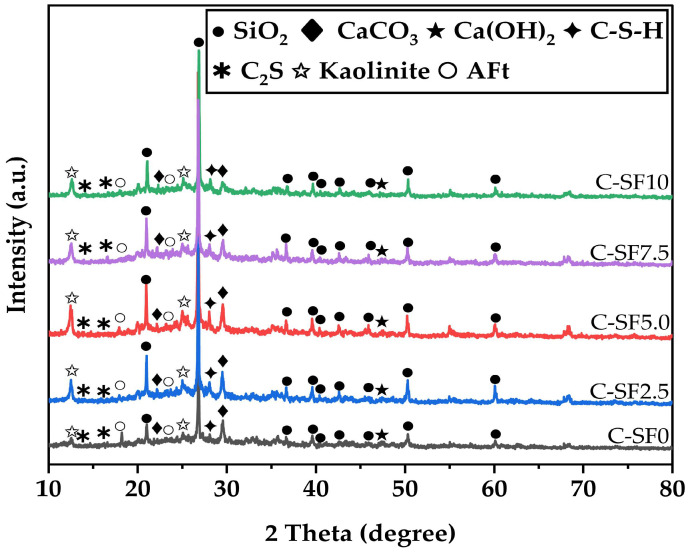
XRD results for SFCP samples at 14 days.

Figure 9 presents the XRD test results for the five groups of samples at 14 days. It can be seen that the main phases of all SFCP samples are the same, including SiO_2_, CaCO_3_, Ca(OH)_2_, C-S-H, C_2_S, Kaolinite, and AFt. However, different SF contents lead to different diffraction peak intensities of the phases. When the SF content is less than 5%, the content of Ca(OH)_2_ gradually decreases and the content of C-S-H gradually increases, which confirms the role of SF in promoting the conversion of Ca(OH)_2_ to C-S-H; when the SF content exceeds 5%, the C-S-H content gradually decreases, which is consistent with the change rule of the UCS. The peak for SiO_2_ is very sharp and its peak intensity is the strongest, which indicates that SiO_2_ mainly exists in the form of crystals in the sample, and its content is high. The peak intensity of CaCO_3_ is markedly diminished, and the peak broadens. This is because the hydration reaction between SF and Ca(OH)_2_ reduces the formation of CaCO_3_, and the particles of SF can effectively fill the pores in the SFCP and reduce the porosity of the material. This decrease in porosity reduces the channeling of CO_2_ into the SFCP, thus slowing down the rate of the carbonation reaction. The increase in the peak width indicates that CaCO_3_ crystals become finer or more dispersed. With a gradual increase in the SF content, the content of C-S-H shows an increasing trend in the range of C-SF0 to C-SF5, and the content of Ca(OH)_2_ decreases gradually. The incorporation of SF facilitates the generation of increased amounts of C-S-H gel, which helps improve the strength and durability of SFCP; this is consistent with the previous experimental results. Some scholars also found that in concrete materials, when 5% SF was used as a variable to replace cement at 7d, the Ca(OH)_2_ content decreased by 10%, 13%, 31%, and 37% in comparison to that in the control group [49].

In addition, when the SF content exceeded 5%, the content of C-S-H showed a gradual downward trend. This is because on the one hand, with a rise in SF replacement and a decrease in the cement content, the content of the Ca element decreases, and the supplementary silicon is insufficient to generate further hydration products with other elements, so the amount of C-S-H gel formed in the same period decreases. The excessive substitution of SF can introduce a large amount of SiO_2_, which participates in the reactions in Equations (9)–(12) during ${\mathrm{SiO}}_{2}{+2H}_{2}{O=\mathrm{Si}(\mathrm{OH})}_{4}$ the hydration process. The generated Si(OH)_4_ and SiO_3_^2−^ can reduce the alkalinity of the activator and affect the formation of the hydration gel [50].

(9) $$S{\mathrm{iO}}_{2}{+2H}_{2}{O=\mathrm{Si}(\mathrm{OH})}_{4}$$

(10) $${\mathrm{Si}(\mathrm{OH})}_{4}+OH^{-}=HSiO_{3}^{-}+2H_{2}O$$

(11) $$HSiO_{3}^{-}+OH^{-}=SiO_{3}^{2-}+H_{2}O$$

(12) $$2HSiO_{3}^{-}=Si_{2}O_{5}^{2-}+H_{2}O$$

#### 3.4.2. Scanning Electron Microscope Image Analysis of SFCP

**Figure 10:**
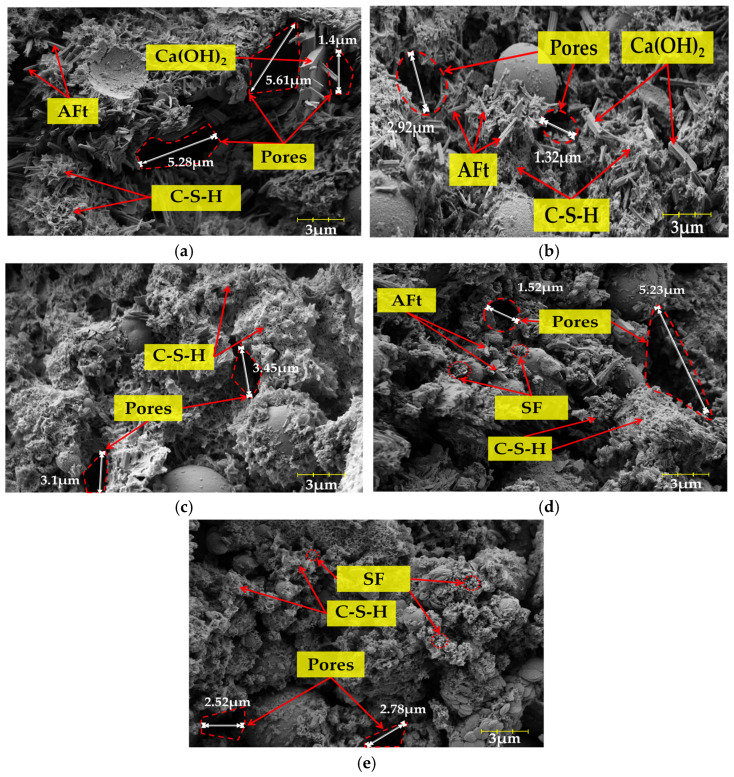
SEM images of SFCP at 14 days of age. (**a**) C-SF0. (**b**) C-SF2.5. (**c**) C-SF5. (**d**) C-SF7.5. (**e**) C-SF10.

Figure 10 presents SEM images of the five groups of samples at 14 days. From Figure 10, we can see that the hydration products mainly include Ca(OH)_2_, hydrated calcium silicate (C-S-H), and calcium vanadate (AFt). In C-SF7.5 and C-SF10, incompletely reacted SF particles can be observed. With an increase in the SF content, the amount of Ca(OH)_2_ gradually decreases, more C-S-H is generated, and the microstructure becomes more compact, which indicates that SF can efficiently facilitate the secondary hydration process of Ca(OH)_2_; this is reflected in the enhancement of the UCS. However, when the content of SF exceeds 5%, there is an agglomeration effect between SF particles, which hinders the further hydration of cement. At the same time, the decrease in the amount of cement directly leads to a decrease in the total amount of Ca^2+^ provided, which leads to an insufficient amount of C-S-H generated by the overall hydration reaction and ultimately leads to a decrease in the UCS. Compared with C-SF0, it can be seen that more C-S-H gel appeared in the C-SF5 group. These hydration products were attached to the surface of FA particles and distributed more evenly, making the microstructure denser and decreasing the observed content of Ca(OH)_2_. This is because the excellent pozzolanic activity of SF accelerates the secondary hydration reaction of Ca(OH)_2_ and transforms it into a denser C-S-H gel, which consumes a large amount of Ca(OH)_2_. Its hydration equation is shown in Formulas (13) and (14) [51]:

(13) $$3CaO\cdot SiO_{2}+{\mathrm{nH}}_{2}O\to xCaO\cdot SiO_{2}\cdot \left(n-3+x\right)H_{2}O+\left(3-x\right)Ca{\left(OH\right)}_{2}$$

(14) $$Ca{\left(OH\right)}_{2}+SiO_{2}+H_{2}O\to CaO\cdot SiO_{2}\cdot H_{2}O(C-S-H)$$

In observations of the images of the C-SF10 group, although the hydration degree of this group seemed to reach a more sufficient level, its UCS showed a downward trend. This is because when the cement content decreases, the amounts of C_3_S and C_2_S also decrease, resulting in a decrease in the amounts of native C-S-H and Ca(OH)_2_ generated during the hydration process. Although SF can increase the rate of formation of C-S-H by a pozzolanic reaction, this reaction depends on the presence of Ca(OH)_2_. As the amount of cement decreases, the amount of Ca(OH)_2_ available for the pozzolanic reaction of SF decreases accordingly. This change leads, in turn, to a decrease in the total amount of C-S-H, which ultimately results in a reduction in compressive strength [52].

From the perspective of the microstructure, most of the pore areas from C-SF0 to C-SF5 were dispersed in the form of small pores, and the hydration products were attached to the pores. The pores were encased by hydration products, and the microstructure was denser. This occurred because SF facilitates Ca(OH)_2_ in establishing a three-dimensional network structure, and C-S-H gel begins to continuously fill the pores as a skeleton to effectively bridge the microcracks between the particles. In addition, due to its smaller particle size, SF can also fill the micropores formed during the hardening process of SFCP samples, thereby refining the cracks and forming a more compact structure, improving the microstructure of the SFCP samples. These conclusions are supported by previous research results (Mahdi Shariati [53]). In the alkaline environment of blast furnace slag, the addition of SF can also increase the formation of C-S-H gel and improve the microstructure.

#### 3.4.3. Image Analysis of Aperture Distribution of SFCP

**Figure 11:**
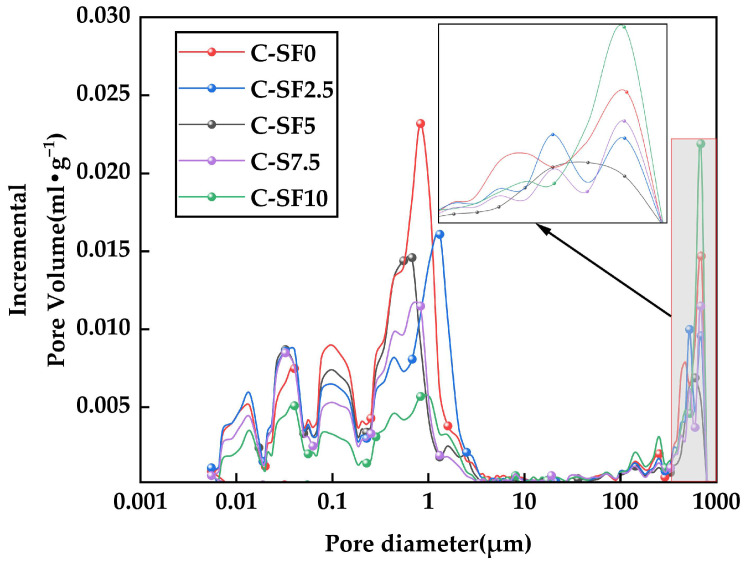
Pore size distribution curve of SFCP.

Figure 11 shows the pore size distribution curve of the SFCP samples at 14 days. By comparing the porosity of the five groups of SFCP, we observed that there were two significant pore size distribution peaks in the five groups of SF samples, which were located in the ranges of 0.01 μm to 1 μm and 100 μm to 800 μm. When SF was added, the significant peaks of the samples near 1 μm and 800 μm were reduced, and the extent of decrease was positively connected with the integration of SF, indicating that the pore structure could be refined by the addition of SF. It is worth noting that in the C-SF10 group, the significant peak near 800 μm was much higher than those in the other groups, which indicates that at this dosage, although SF reduces the size of small pores, it also hinders the hydration reaction of cement, resulting in the formation of more large pores. This is in line with the macroscopic change law of the UCS. In the pore size range of 0.1 μm to 1 μm, the number of microcracks less than 1 μm in the sample group with SF was significantly lower than that in C-SF0 (control group). This indicates that the fine particles of SF can effectively fill the tiny pores formed during the hydration process of SFCP samples, thereby producing a denser structure and reducing the number of microcracks. The addition of an appropriate amount of SF can promote the secondary hydration reaction of cement, leading to the augmentation of hydration products such as C-S-H and AFt. These products are intertwined to form a dense network structure, which significantly reduces the pores between particles [54], thereby effectively reducing the large cracks in the range of 100 μm to 800 μm. In particular, the porosity of C-SF10 was significantly lower than that of other groups in the range of less than 1 μm, but it was much higher than that of other groups in the vicinity of 800 μm. This phenomenon is due to the excessive addition of SF. Although the addition of SF largely fills the tiny pores in the developed cracks, when the cement is replaced by a large amount, the amount of Ca element in the SFCP is significantly reduced and cannot produce enough Ca(OH)_2_, which further leads to a decrease in the number of hydration products such as C-S-H. Although the incorporation of SF can contribute to physical filling, excessive SF may hinder the hydration reaction of the cement itself, causing greater porosity and a rougher microstructure. When SFCP is compressed, stress concentration occurs around the pores, and these stress concentration areas are likely to cause local damage, thereby reducing the overall UCS. In prior experiments, researchers such as Kim [55] successfully optimized the porosity by incorporating nano-silica and SF into concrete. In addition, they also found that when the particle size of the incorporated particles is more delicate, the optimization effect on the porosity is more significant.

It is worth noting that the porosity of C-SF5 actually exceeded that of C-SF2.5 at a scale of 0.1 μm, which may be attributed to the formation of Thaumasite (TSA). Thaumasite has been demonstrated to be an expanding hydration product that easily causes microcracks in SFCP [56]. Under certain conditions, C-S-H gel reacts with the AFt phase, and the Si element in the C-S-H gel gradually replaces the aluminum element in the AFt phase. As the reaction continues, the AFt phase is finally transformed into TSA; the related chemical reaction is shown in Equation (15).

(15) $$\begin{array}{l} Ca_{6}{\left[Al_{x}Fe_{\left(1-x\right)}{\left(OH\right)}_{6}\right]}_{2}{\left(SO_{4}\right)}_{3}\cdot 26H_{2}O+Ca_{3}Si_{2}O_{7}3H_{2}O+2CaCO_{3}+4H_{2}O\\ \to Ca_{6}{\left[Si{\left(OH\right)}_{6}\right]}_{2}{\left(CO_{3}\right)}_{2}{\left(SO_{4}\right)}_{2}24H_{2}O+CaSO_{4}2H_{2}O+2xAl{\left(OH\right)}_{3}\\ +2\left(1-x\right)Fe{\left(OH\right)}_{3}+4Ca{\left(OH\right)}_{2} \end{array}$$

The reduction in pore size and densification of the microstructure not only enhance the mechanical strength of the material but also significantly improve its resistance to chemical penetration and other environmental factors, primarily through the optimization of the pore structure [57] and the promotion of calcium silicate hydrate (C-S-H) gel formation. This improvement in microstructure is particularly critical under highly variable underground conditions. The decrease in the total porosity and the refinement of the pore size distribution effectively minimize the pathways for aggressive agents such as chloride ions, sulfate ions, and moisture [58], which are the primary causes of material degradation in underground environments. By reducing the risk of exposure to corrosive substances, SFCP materials with an optimal SF content can significantly enhance the long-term durability of backfill operations, reduce maintenance requirements, and mitigate the risk of material degradation. This contributes to achieving long-term sustainability and lowering environmental impacts during continuous mining backfill processes.

## 4. Conclusions

In this study, SFCP was prepared by replacing part of the cement in FCP with SF, and the fluidity, uniaxial compressive strength, microstructure, and pore structure of the SFCP were analyzed. The main conclusions are as follows:

1. The incorporation of silica fume had a significant impact on the setting time of the slurry. When the silica fume content was 5%, the initial and final setting times of the slurry were 900 min and 980 min, respectively, representing reductions of 6.25% and 7.5% compared to the control group. However, when the silica fume content exceeded 5%, the setting time increased significantly, which is detrimental to improving construction efficiency.
2. As the silica fume content increased, the flowability of the slurry gradually decreased. Notably, when the silica fume content exceeded 7.5%, the flowability dropped sharply, with the spread decreasing by 67% and the slump decreasing by 16% compared to those for the control group, though it still met the requirements for pipeline transportation. A moderate incorporation of silica fume (≤5%) can enhance material performance while maintaining basic flowability.
3. The silica fume content had a significant effect on the uniaxial compressive strength (UCS) of the slurry. At a curing age of 14 days, the UCS reached 3.98 MPa when the silica fume content was 5%, representing a 25% increase compared to the control group, indicating optimal medium-term strength. However, when the silica fume content exceeded 5%, the UCS decreased significantly, with a maximum reduction of 41% due to particle agglomeration and restricted hydration reactions. A moderate silica fume content promotes the formation of C-S-H gel through secondary hydration reactions, enhancing structural strength, while excessive silica fume disrupts this balance, leading to a strength reduction.
4. The microstructural analysis revealed that the addition of an appropriate amount of silica fume significantly improved the slurry’s density, reduced the number and distribution of micropores, and optimized the pore structure. At a silica fume content of 5%, the formation of C-S-H gel was maximized, and the pore structure was most optimized. However, high silica fume contents (≥7.5%) resulted in particle agglomeration and the formation of large pores, which weakened the overall strength and durability of the material. This indicates that the silica fume content must be carefully controlled to balance its positive and negative effects.
5. Considering the effects of silica fume on the setting time, flowability, compressive strength, and microstructure, the optimal silica fume content was determined to be 5%. This content maintains material flowability while significantly improving the medium-term strength and structural density. This finding provides a scientific basis for the design of backfill materials under continuous mining and backfilling conditions.

## Figures and Tables

**Table 1:** Main chemical composition of materials (wt%).

Chemical Composition	CaO	SiO_2_	Fe_2_O_3_	Al_2_O_3_	K_2_O	MgO	SO_3_	Na_2_O
SF	0.49	90.36	0.08	0.44	0.52	1.03	1.42	0.46
OPC	64.78	20.34	3.11	5.02	0.35	1.09	2.20	0.10
FA	8.11	43.90	12.91	27.42	0.95	2.01	2.11	1.02
Gangue	3.38	55.50	5.44	18.15	1.67	1.23	0.64	0.64

**Table 2:** Experimental conditions.

No.	Item	Solid Content [wt%]	Cement [wt%]	SF [wt%]	FA [wt%]	Water [g]
1	C-SF0	76	12.0	0.0	38	554
2	C-SF2.5	76	9.5	2.5	38	554
3	C-SF5	76	7.0	5.0	38	554
4	C-SF7.5	76	4.5	7.5	38	554
5	C-SF10	76	2.0	10.0	38	554

**Table 3:** Fitting results of rheological parameters of SFCP slurry with different SF contents.

No.	SF Content [%]	τ0	K [Pa]	n	Fitting [R^2^]
C-SF0	0	35.97	0.0468	1.979	0.9924
C-SF2.5	2.5	40.32	0.0240	1.855	0.9886
C-SF5	5	60.45	0.0225	2.016	0.9805
C-SF7.5	7.5	100.1	0.0166	2.124	0.9801
C-SF10	10	138.6	0.0117	2.239	0.9752

**Table 4:** ANOVA summary.

Curing Time [days]	SSB	SSW	MSB	MSW	F	P
3	0.4486	0.0691	0.1122	0.0069	16.22	0.00023
7	6.4211	0.1519	1.6053	0.0152	105.71	3.87 × 10^−8^
14	7.5415	0.2454	1.8854	0.0245	76.83	1.82 × 10^−7^
28	8.9655	0.1046	2.2414	0.0105	214.18	1.21 × 10^−9^

## Data Availability

The data used to support the findings of this study are included in the article.

## Abbreviations

FA	Fly ash
SF	Silica fume
FCP	Fly ash cement paste
SFCP	Silica fume cementitious paste
CMCB	Continuous mining and continuous backfilling
C-SF	The proportion of cement replaced by an equivalent amount of silica fume
τ_0_	Yield stress
*K*	Rheological consistency index
*n*	Rheological flow behavior index
*R* ^2^	Coefficient of determination (goodness of fit)
UCS	Uniaxial compressive strength
C-S-H	Calcium silicate hydrogel
XRD	X-ray diffraction
SEM	Scanning electron microscope
MIP	Mercury intrusion porosimetry
AFt	Alumina ferrite trisulfate
$\bar{X}$	Overall sample mean
${\bar{X}}_{i}$	Mean of the *i*-th group
$X_{ij}$	Observation *j* in group *i*
*N*	Total number of observations
*k*	Number of groups
*n_i_*	Number of observations in the *i*-th group
*SSB*	Sum of squares between groups
*SSW*	Sum of squares within groups
*MSB*	Mean square between groups
*MSW*	Mean square within groups
*df_between_*	Degrees of freedom between groups
*df_within_*	Degrees of freedom within groups
*F*	F-statistic for ANOVA
*P*	*p*-value for significance testing
